# Comparative gene expression profiling of muscle reveals potential candidate genes affecting drip loss in pork

**DOI:** 10.1186/s12863-019-0794-0

**Published:** 2019-12-02

**Authors:** Xueyan Zhao, Cheng Wang, Yanping Wang, Haichao Lin, Huaizhong Wang, Hongmei Hu, Jiying Wang

**Affiliations:** Shandong Provincial Key Laboratory of Animal Disease Control and Breeding, Institute of Animal Science and Veterinary Medicine, Shandong Academy of Agricultural Sciences, Jinan, 250100 China

**Keywords:** Pig, Meat quality, RNA sequencing, Differentially expressed genes

## Abstract

**Background:**

Drip loss is a key aspect of meat quality. Transcriptome profiles of muscle with divergent drip loss would offer important insight into the genetic factors responsible for the trait. In this study, drip loss and other meat quality traits of 28 purebred Duroc pigs were measured, muscles of these individuals were RNA sequenced, and eight individuals with extremely low and high drip loss were selected for analyzing their transcriptome differences and identifying potential candidate genes affecting drip loss.

**Results:**

As a result, 363 differentially expressed (DE) genes were detected in the comparative gene expression analysis, of which 239 were up-regulated and 124 were down-regulated in the low drip loss group. The DE genes were further filtered by correlation analysis between their expression and drip loss values in the 28 Duroc pigs measured and comparison of them with QTLs affecting drip loss. Consequently, of the 363 DE genes, 100 were identified as critical DE genes for drip loss. Functional analysis of these critical DE genes revealed some GO terms (extracellular matrix, cell adhesion mediated by integrin, heterotypic cell-cell adhesion), pathway (ECM-receptor interaction), and new potential candidate genes (*TNC, ITGA5*, *ITGA11*, *THBS3* and *CD44*) which played an important role in regulating the variation of drip loss, and deserved to carry further studies to unravel their specific mechanism on drip loss.

**Conclusions:**

Our study revealed some GO terms, pathways and potential candidate genes affecting drip loss. It provides crucial information to understand the molecular mechanism of drip loss, and would be of help for improving meat quality of pigs.

## Background

Meat quality can be evaluated by multiple indicators, such as water holding capacity (WHC), meat color, intramuscular fat (IMF) and tenderness. Among these, WHC, i.e. the ability of meat to retain water, is arguably one of the most important commercially interesting indicators, which directly influences the appearance, sensory quality, and the value of meat produced. WHC is often predicted by drip loss, which is defined as the fresh meat loss rate under gravity at 4 °C for 24 h [1]. Moreover, WHC is closely related to other meat quality indicators, including meat color, pH, and IMF [2, 3].

Large phenotypic differentiation of drip loss in musculus longissimus dorsi (LD) was observed in the previous studies [4, 5]. Apart from environmental factors, this wide range could be explained by genetic factors which directly affect the muscle characteristics.Therefore, it is considered as a target trait in some breeding schemes. However, drip loss exhibits low to moderate heritability, varying from 0.08 to 0.30 depending on the measurement methods or breeds [6–8], and it is difficult to improve such traits using conventional breeding methods. Therefore, the development of valuable molecular markers or the identification of key genes related to drip loss would provide new opportunities for decreasing drip loss of pork using molecular breeding technologies.

Drip loss, like the other meat quality traits, is a complex quantitative trait and influenced by many genes. Until now, a variety of genetic approaches have been applied to elucidate the genetics that underlies this trait, such as identifying quantitative trait loci (QTLs), candidate genes and other markers. To date, 1115 QTLs related to WHC and drip loss (http://www.animalgenome.org/cgi-bin/QTLdb/SS/index, Release 39, Aug 22, 2019) were reported in different pig populations. However, these QTLs were identified mostly via genome scan based on microsatellite genotyping, and generally large genomic regions. Further fine-mapping studies to find causal genes are challenging. Recently, with the development of high-density SNP chips and genome sequencing, genome-wide association studies (GWAS) have been used as a more precise method for identifying the genomic regions and markers associated with drip loss [9–11]. Moreover, in the studies of candidate genes, two major genes, *RYR1* [12] and *PRKAG3* [13], have been demonstrated to control drip loss for pale, soft and exudative (PSE) and dark, firm and dry (DFD) meat, respectively, and one causal gene, *PHKG1* [14], for normal quality meat. Despite these progress, the knowledge of the genetic factors underlying drip loss remains very incomplete.

Gene expression patterns may explain a high degree of the observed phenotypic differences in a given tissue. Transcriptomics, based on RNA sequencing (RNA-seq), enables high throughput screening of expressed genes, and is a powerful tool for exploring the genetic architecture of complex traits. Previous studies examining the transcriptome of the porcine LD muscle have successfully identified differentially expressed (DE) genes affecting backfat thicknesses [15], IMF [16] and drip loss [17, 18]. Although some DE genes have been identified, the results are not consistent among studies. In this study, the LD muscles of individuals with extremely low and high drip loss were sampled from a purebred Duroc pig population, and RNA-seq technology was applied to screen DE genes, followed by functional analysis to identify plausible candidate genes affecting drip loss. This study provides valuable information for understanding the molecular basis underlying drip loss of pigs.

## Results

### Drip loss variation among Duroc pigs

Twenty-eight Duroc pigs were used in this study. Their detailed information of carcass and meat quality traits is presented in Table 1. It can be seen that these pigs had good carcass quality, and their lean meat rate, backfat thickness and large eye muscle area were 66.90%, 16.30 mm and 46.94 cm^2^ in average, respectively. And these pigs also showed to have normal meat quality according to the traits of pH_1_, pH_24_ as well as L*, a* and b*, whose averages were 6.49, 5.72, 42.78, 5.72, and 10.63, respectively.

**Table 1:** Phenotypic information of drip loss and other carcass and meat quality traits

Traits	Total 28 samples measured	Four samples of Low drip loss group	Four samples of High drip loss group	*P* value
Drip loss (%)	2.36 ± 0.53	1.69 ± 0.06	3.03 ± 0.09	0.002
Weight (kg)	108.30 ± 6.00	107.3 ± 1.68	107.5 ± 2.83	0.285
Backfat (mm)	16.30 ± 4.49	14.6 ± 3.68	15.1 ± 2.12	0.198
Eye muscle area (cm^2^)	46.94 ± 5.38	53.8 ± 3.84	50.2 ± 1.84	0.107
Percentage of lean meat (%)	66.90 ± 2.57	68.9 ± 2.47	69.1 ± 2.05	0.366
pH_1_	6.49 ± 0.22	6.39 ± 0.09	6.79 ± 0.01	0.053
pH_24_	5.72 ± 0.14	5.79 ± 0.11	5.67 ± 0.02	0.078
IMF (%)	2.36 ± 0.71	2.05 ± 0.48	2.45 ± 0.17	0.264
Shear force(N)	45.80 ± 10.96	59.8 ± 10.26	44.85 ± 7.85	0.025
L*	42.78 ± 1.82	43.09 ± 1.14	43.93 ± 0.74	0.590
a*	5.72 ± 0.78	4.94 ± 0.34	5.94 ± 0.30	0.027
b*	10.63 ± 0.80	10.27 ± 0.41	11.16 ± 0.04	0.407

The results are showed by mean ± standard deviation

Specifically for the values of target trait, drip loss, they were normally distributed with *P* value of 0.1787 for Shapiro-Wilk normality test, and the average was 2.36%, with the minimum and maximum of 1.63 and 3.67%, respectively. Four individuals with extremely low drip loss (2 male and 2 female) and four with extremely high drip loss (2 male and 2 female) were selected for further genome-wide gene expression analysis. No sib or half-sib relationship existed among the 8 individuals selected for the transcriptome analysis.

**Fig. 1:**
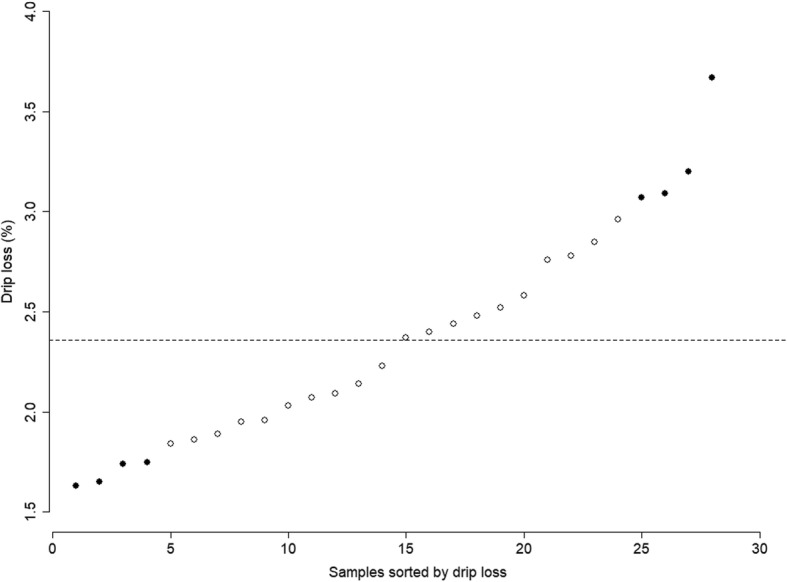
Distribution of the low and high drip loss samples selected for RNA-seq in the 28 Duroc pigs. The solid dots indicate the samples selected for RNA-seq

The detailed phenotypic information of the eight individuals is provided in Additional file 1: Table S1 and Table 1. And the distribution of drip loss among the 28 individuals is demonstrated in Fig. 1. Compared between the two groups, the average of the high drip loss group (3.1%) was significantly greater than that of the low drip loss group (1.69%) with *P* value of 0.002. It is important to note that besides drip loss, shear force, and a* also show significant differences between the two groups, demonstrating that there are close correlations between drip loss and these traits.

### Summary statistics for RNA-seq data

Using paired-end RNA-seq approach, we sequenced the transcriptome of LD muscle of the 28 Duroc pigs. In total, we generated 52.43–86.93 million paired-end raw reads per sample. Prior to assembly, low quality reads, which was 2.31% of the raw reads, were removed. As a result, an average of 62.56 million cleaned reads were produced for the 28 samples after filtering. A summary of the sequencing results is shown in Additional file 2: Table S2. After alignment, an average of 90.36 and 4.01% of the cleaned reads could be mapped to the recent *Sus scrofa* genome (Sscrofa 11.1) with unique position and multiple positions respectively. Additionally, out of the reads mapped to the reference genome, 91.58% fell in annotated exons, 3.86% were located in introns, and the remaining 4.56% were assigned to intergenic regions. Unmapped or multiposition matched reads were excluded from further analyses.

### Expression profiling of muscle with low and high drip loss

A total of 26,918 genes were detected for samples sequenced through alignment with the recent *Sus scrofa* genome. After filtering those genes with a minimal number of reads (≤1) in more than one third of the 28 individuals, there were 18,064 genes expressed, whose normalized expression values by DESeq2 is presented in Additional file 3: Table S3. And further analyses were conducted based on them. Out of the 18,064 genes, 17,071 were included in Ensembl pig genome database, while the other 993 were unannotated in *Sus scrofa* reference genome (Sscrofa 11.1) and potential novel genes (Additional file 4: Table S4).

**Fig. 2:**
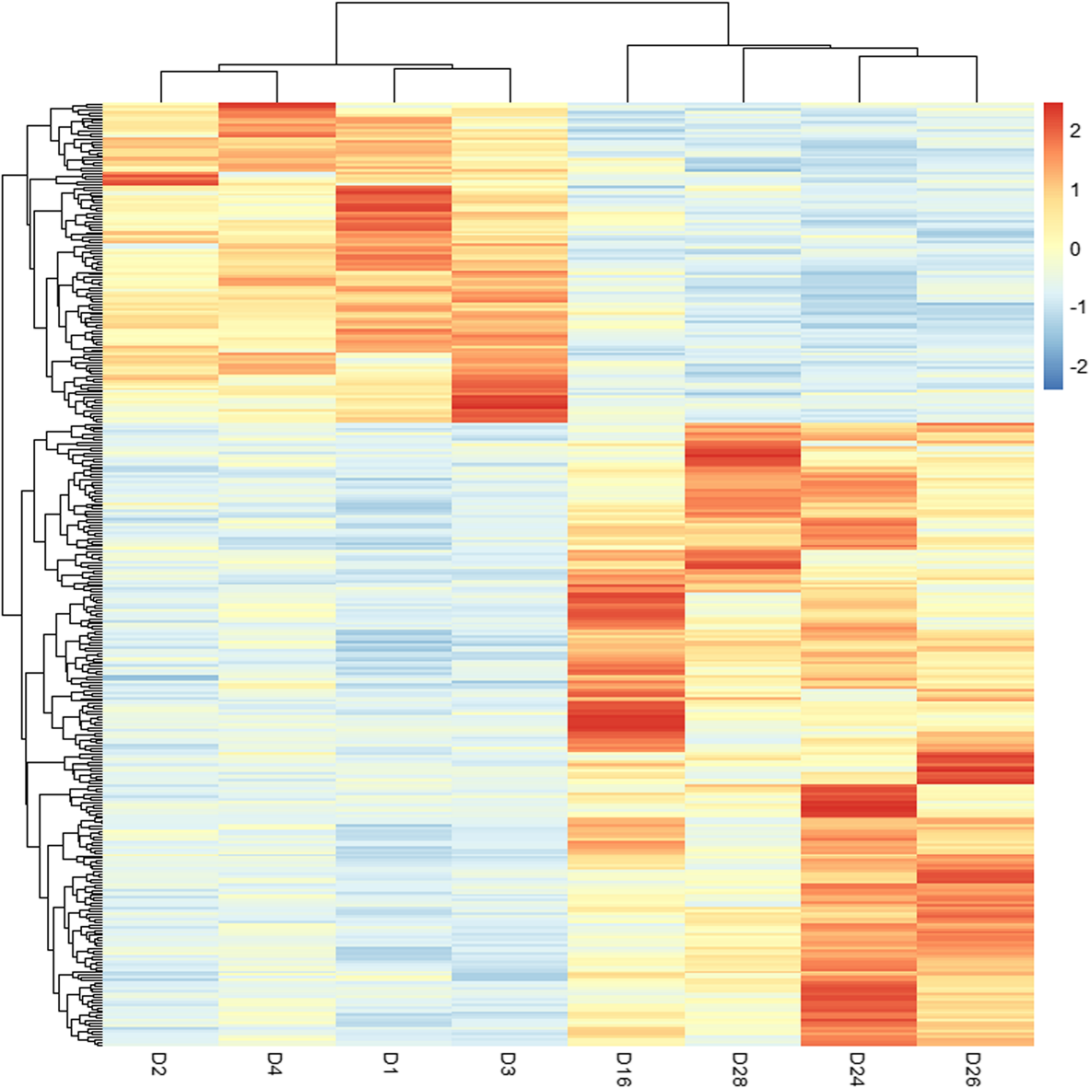
Heatmap for DE genes. Columns and rows indicate pigs and DE genes, respectively. Color scale represents log_10_(normalized expression values of DE genes). Red and blue represent up- and down-regulated DE genes, respectively. DE genes, differentially expressed genes

Using DESeq2, the differential expression analysis between low and high drip loss groups was performed. According to the empirical studies, FDR adjustment of *P* value < 0.05 and fold change ≥2 or fold change < 0.5, were used to reduce false positive rate of DE genes. As a result, a total of 363 genes showed significant expression differences between the two groups, and 239 and 124 genes were up-regulated and down-regulated in the low drip loss group, respectively. Their detailed information is provided in Additional file 5: Table S5. Figure 2 shows the heatmap of these DE genes, from which, it can be seen that the expression patterns are consistent within groups and different between groups.

### Correlation between expression of DE genes and their drip loss values

**Fig. 3:**
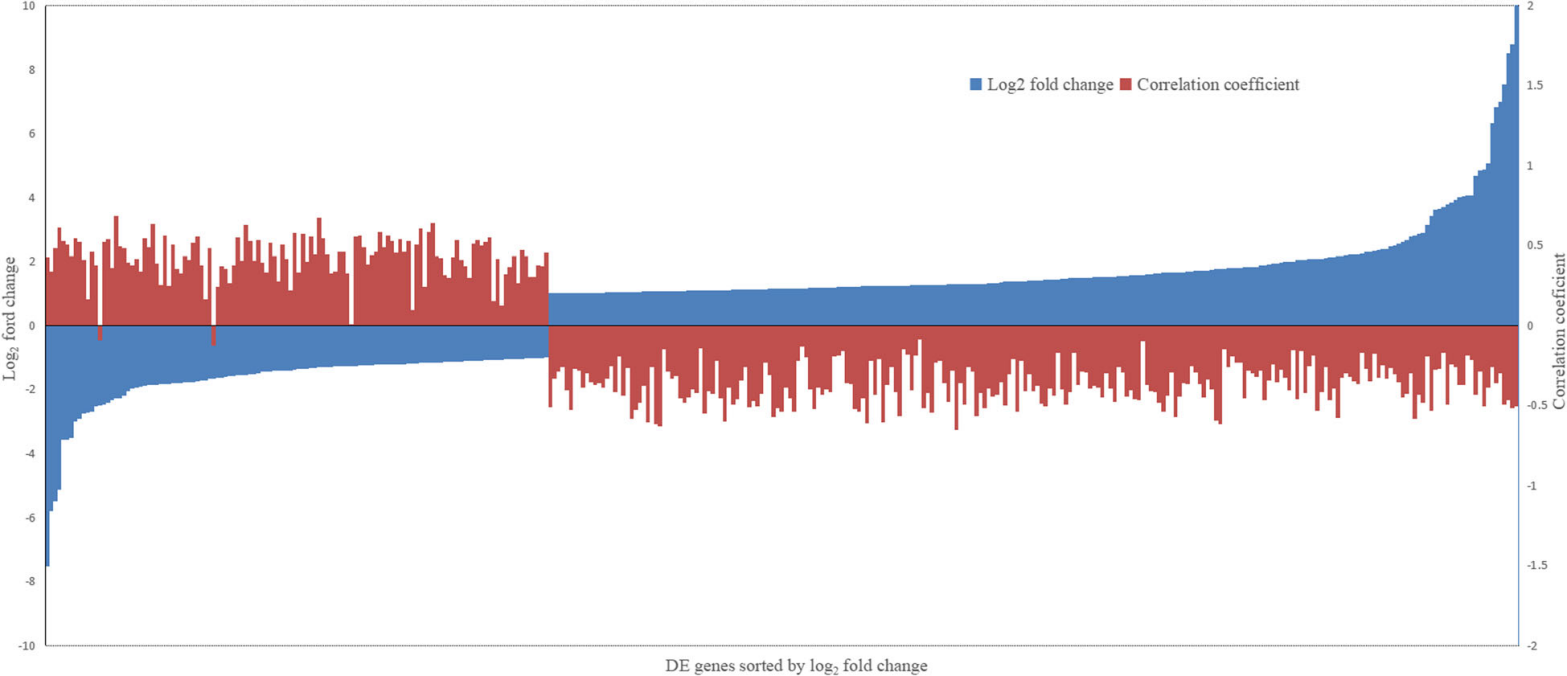
Pearson’s correlation coefficients between expressions of DE genes detected and their drip loss values in the 28 Duroc pigs measured in this study. DE genes, differentially expressed genes

To filter the DE genes detected, Pearson correlation analysis was conducted between expressions of these DE genes and their drip loss values in the data set of 28 Duroc pigs measured in this study. As shown in Fig. 3, expressions of all the up-regulated DE genes were negatively correlated with their drip loss (correlation coefficients ranging from 0.00 up to 0.69), while those of all but two of the down-regulated DE genes were positively correlated (correlation coefficients ranging from − 0.65 up to − 0.09). Furthermore, significance test of correlation analysis indicated that there were 209 genes, including 122 down-regulated and 87 up-regulated genes, whose expressions and the drip loss values were significantly correlated (*p* < 0.05). The detailed information of these significantly correlated DE genes is presented in Additional file 6: Table S6.

### Comparison of DE genes with QTLs affecting drip loss

We further filtered the DE genes detected by comparing the DE genes with the QTLs affecting drip loss collected in the pig QTL database (http://www.animalgenome.org/cgi-bin/QTLdb/SS/index, Release 39, Aug 22, 2019). In total, 1115 QTLs in the pig QTL database were reported to relate with drip loss or WHC. Since some QTLs have identical physical positions or no exact physical position, 310 QTLs with unique physical position were used to compare with DE genes. As a result, 192 DE genes were mapped to the 60 of these 310 QTLs (Additional file 7: Table S7). Further enrichment analysis by Fisher’s exact test indicated that DE genes were significant enriched in QTLs affecting drip loss (*P value* < 2.2e-16). High proportion of DE genes mapping in QTLs affecting the trait proofed that the DE genes detected in the study were more likely to be candidate genes affecting drip loss.

### Functional analysis of critical DE genes detected for drip loss

Combining the above two filtering conditions, of the 363 DE genes, we identified 100 critical DE genes for drip loss (Additional file 8: Table S8). They located in the QTLs related to drip loss, and their expressions were significantly correlated with drip loss values in 28 Duroc pigs. To have a functional view of them, we carried out Gene Ontology (GO) and Kyoto Encyclopedia of Genes and Genomes (KEGG) enrichment analysis for them, and determined 12 significantly enriched GO terms (Additional file 9: Table S9) and three pathways (Additional file 10: Table S10) with *P* value < 0.01. Among the 12 enriched GO terms, 10 terms were belonged to biological process (BP) category and involved in cell adhesion, wound healing and amino acid transport, meanwhile the other ones, cell surface and extracellular matrix, were belonged to cellular component (CC) category.

**Fig. 4:**
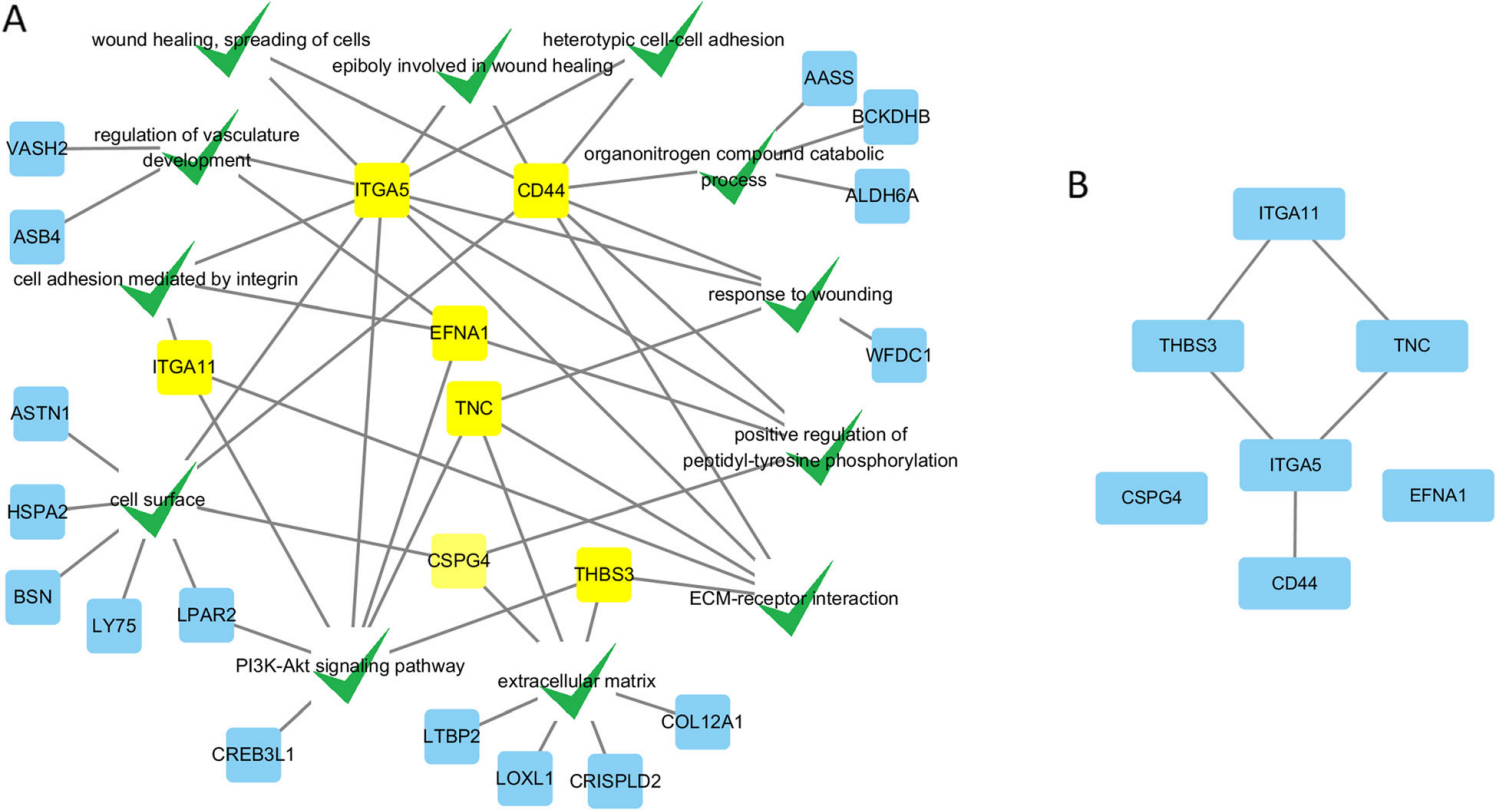
**a** some critical DE genes and their belonged GO terms and pathways. Green ticks represent the enriched GO terms and pathways. Squares represent the critical DE genes, of which yellow ones represent the seven critical DE genes involved in more than three GO terms or pathways. GO, Gene Ontology; DE genes, differentially expressed genes. **b** protein-protein interaction (PPI) network of the seven critical DE genes involved in more than three GO terms or pathways

Figure 4 illustrates part of critical DE genes involved in the nine significantly enriched GO terms and three pathways which may related to drip loss. Among these genes, *TNC* (*q* value = 2.90E-03, FC = 2.98), *ITGA5* (*q* value = 4.96E-03, FC = 2.35), *ITGA11* (*q* value = 7.88E-04, FC = 2.17), *CD44* (*q* value = 2.77E-04, FC = 2.35), *EFNA1* (*q* value = 2.61E-02, FC = 2.14), *CSPG4* (*q* value = 4.05E-07, FC = 2.45), and *THBS3* (*q* value = 2.27E-07, FC = 2.35) were involved in more than three GO terms or pathways, implying there could be possible interactions among these genes. We further constructed protein-protein interaction (PPI) network analysis using Search Tool for the Retrieval of Interacting Genes (STRING). Consequently, a PPI network, consisting of five core proteins *TNC, ITGA5*, *ITGA11*, *THBS3* and *CD44,* was constructed, suggesting the five genes biologically connected (Fig. 3). Table 2 presents the detailed information of the five candidate genes.

**Table 2:** List of some potential candidate genes between low and high drip loss groups

Gene information		Differential expression values between two groups		Pearson correlation in 28 Duroc pigs		QTL_ID	Gene description
Ensembl_ID	Gene name	Log_2_ fold change	FDR adjusted *P* value	Correlation coefficient	*P* value		
ENSSSCG00000000293	*ITGA5*	2.19	7.23E-05	−0.4754	1.06E-02	3805, 9847	Integrin subunit alpha 5
ENSSSCG00000004961	*ITGA11*	2.17	5.20E-06	−0.4936	7.60E-03	7936, 7784	Integrin subunit alpha 11
ENSSSCG00000013297	*CD44*	2.35	1.20E-06	−0.4341	2.10E-02	86, 2937, 3780, 21,848, 38,075	CD44 molecule
ENSSSCG00000005494	*TNC*	2.98	3.32E-05	−0.4659	1.24E-02	7936, 8419	Tenascin C
ENSSSCG00000006524	*THBS3*	2.35	2.27E-07	−0.6102	5.65E-04	3847, 38,084	Thrombospondin 3

## Discussion

Understanding the molecular mechanisms underlying drip loss is a essential way to improve the economic value of pork. In this study, to identify candidate genes influencing drip loss of pigs with normal meat quality, we selected extremely low and high drip loss samples from a high number of Duroc pigs with the same environment, performed a comparative expression profiling analysis, and detected 363 DE genes (Additional file 5: Table S5) between the two groups with extremely low and high drip loss. The DE genes were further filtered by correlation analysis between their expression and drip loss value in the 28 Duroc pigs measured and comparison of them with QTLs affecting drip loss to reduce false positive. Consequently, we identified 100 critical DE genes affecting drip loss (Additional file 8: Table S8), which located in the QTLs related to drip loss, and whose expressions were significantly correlated with drip loss values in 28 Duroc pigs. These critical DE genes would provide crucial information to understand the molecular mechanisms underlying drip loss.

Some enriched GO terms and pathways related to extracellular matrix, such as extracellular matrix (GO:0031012, extracellular matrix) and ECM-receptor interaction (KEGG:ssc04512, ECM-receptor interaction), were identified in the functional analysis of the critical DE genes detected. Extracellular matrix consists of many proteins, which interact with each other and form a super molecular network to withstand and transmit the contractile forces generated by muscle fibers [19], and thus may play important role in influencing drip loss. Besides extracellular matrix, integrins have been proposed to attach the cytoskeleton to the extracellular matrix, and have impact on the formation of drip channels in pork [20]. Previous works have indicated that there was a reverse correlation between postmortem degradation of integrins and drip loss [21, 22]. In our result, two enriched GO terms related to integrins and adhesion, cell adhesion mediated by integrin (GO:0033627) and heterotypic cell-cell adhesion (GO:0034113), were also discovered. Therefore, extracellular matrix, cell adhesion mediated by integrin and heterotypic cell-cell adhesion are potential candidate GO terms, while ECM-receptor interaction are potential candidate pathway affecting drip loss.

Among the critical DE genes detected in the study, *TNC, ITGA5*, *ITGA11*, *THBS3* and *CD44* were not only repeatedly discovered as the important genes in the enriched GO terms and pathways (Fig. 4), but coded the five core proteins in the PPI network analysis. *TNC* encodes an extracellular matrix glycoprotein, interacts with several other extracellular matrix molecules and cell-surface receptors, thus affecting tissue architecture, tissue resilience and cell responses. It is significantly up-regulated in response to tissue injury [23]. The product of *ITGA5* and *ITGA11* belong to the integrin alpha chain family. Integrins are heterodimeric integral membrane proteins composed of an alpha subunit and a beta subunit that function in cell surface adhesion and signaling [24]. *THBS3* is an adhesive glycoprotein that is involved in cell-cell and cell-matrix interactions. Newly study by Schips [25] demonstrated that *THBS3* promotes sarcolemmal destabilization by reducing integrin function, augmenting disease-induced decompensation. *CD44* is a multifunctional transmembrane glycoprotein that serves as a cell-surface receptor for a number of ECM proteins [26]. It can be seen that the main function of the five genes are related to extracellular matrix and integrins, which as mentioned above, have impact on the formation of drip loss. All the above proofs that the five genes are potential candidate genes affecting drip loss.

Except potential candidate genes listed above, we also confirmed several known candidate genes influencing drip loss reported in the previous studies. Heat shock proteins (HSPs) HSPs belong to stress related proteins, and have been reported as potential biomarkers for several meat quality traits (WHC, tenderness, and colour) [27, 28]. In this study, several HSP genes, like *HSPB1*, *HSPH1*, *HSPB7* and *HSPA1L* were found to not only differently express between the low and high drip loss groups, but significantly correlated in the correlation analysis in the 28 Duroc pigs. An improved abundance of these proteins may enable cells to overcome stressful conditions and leads to less fluid exuding from the cells. In addition, genes of solute carrier family (SLC), such as *SLC37A4* and *SLC3A2,* have been reported to be promising candidate genes influencing drip loss trait [18, 29]. In our study, *SLC37A4* was differently expressed between divergent drip loss groups, significantly correlated in the large samples, and located in the QTL (QTL_ID 3806) related to drip loss. Hence, our study confirmed it to be a promising candidate gene for drip loss. Additionally, we identified several other genes of solute carrier family members, suggesting the importance of gene of solute carrier family in regulating drip loss.

## Conclusion

In this study, transcriptome analysis based on RNA-seq technology was performed to evaluate the gene expression differences of muscle with divergent drip loss. A total of 363 DE genes were screened by DESeq2 with 239 and 124 genes up-regulated and down-regulated in the low drip loss group, respectively. Combining the filtering conditions of correlation analysis and comparison with QTLs related to drip loss, we identified 100 critical DE genes affecting drip loss from the 363 DE genes detected. Further functional analysis of the critical DE genes revealed some GO terms (extracellular matrix, cell adhesion mediated by integrin, heterotypic cell-cell adhesion), pathway (ECM-receptor interaction), and new potential candidate genes (*TNC, ITGA5, ITGA11, THBS3 and CD44*) which have impact on the variation of drip loss.

## Methods

### Animals and phenotypic traits

Thirteen male and 15 female purebred Duroc pigs from Jianghai Pig Breeding Co., Ltd. were used in our study. The pigs came from different sires and dams, and thus no sib and half-sib relationships were existed among them. The animals were reared indoors in a standard pig breeding farm and fed with diets formulated according to age under a standardized feeding regimen with free access to water. All pigs were slaughtered at an average weight of 108.29 ± 6.00 kg (mean ± standard deviation) in one batch in compliance with national guidelines. The pigs were stunned by electric shock, and the carcasses were exsanguinated, scalded and split at unconsciousness. Moreover, all pigs were free of the Halothane gene mutation (RYR1; C1843T), ensured by DNA testing prior to the experiment.

After slaughter, the backfat thickness (average thickness of the thickest of shoulder, the last rib and the lumbosacral junction) and eye muscle area (at the last rib) were measured using a vernier caliper. After splitting the left carcass into lean meat, fat, skin and bone, the percentage of lean meat was estimated by calculating the lean meat percentage of the sum of lean meat, fat, skin and bone. The meat quality traits were evaluated using samples taken from LD muscle of the left carcass. Specifically, muscle pH at 45 min postmortem (pH_1_) and 24 h postmortem (pH_2)_ were measured at the last 1st and 2nd thoracic vertebrae using a portable pH meter (MATTHAUS PH-STAR, German) for meat. Drip loss was measured in triplicate per sample using three muscle samples about 10 g taken from the last 3rd and 4th thoracic vertebra within 60 min postmortem. Each one was weighed and placed into a plastic measuring device like a funnel with lid. After storage for 24 h at 4 °C, the samples were dabbed with filter paper and reweighed. Drip loss was calculated as the percentage of weight loss based on the initial weight of the sample. Muscle sample was taken from the last 4th and 5th thoracic vertebrae, trimmed of external fat, minced, and dried for determination of IMF content using chloroform-methanol extraction method. IMF content was measured in triplicate per sample to ensure its accuracy. The CIE L* (lightness), a* (redness), and b* (yellowness) parameters were determined at the 1st lumbar vertebra within 60 min postmortem using a CR310 Minolta chromameter (Osaka, Japan). Shear force was also determined using samples taken from the 1st and 3rd lumbar vertebrae. Samples were firstly aged up to 4 d postmortem at 4 °C, then heated in 80 °C water-bath until the center temperature up to 70 °C. After cooled at room temperature, samples parallel to fiber axis were cut into 1 cm^2^ sections for measurement of shear force perpendicularly to muscle fibers using a digital muscle tenderness meter (C-LM3B, Northeast Agricultural University, Harbin, China). Shear force was the average of 15 measurements per sample.

### Total RNA extraction, library construction and sequencing

Immediately after slaughter, LD muscles were taken from the 4th thoracic vertebra from the last rib, put into tubes with RNAlater Stabilization Solution (Thermo Fisher, Waltham, MA, USA), and frozen at − 80 °C for RNA extraction. Total RNA was isolated from the 28 samples using TRIzol reagent (Invitrogen, Life Technologies). The purity and concentration of total RNA were assessed by NanoDrop 1000 instrument (Thermo Fisher Scientific),) and Qubit® RNA Assay Kit in Qubit® 2.0 Flurometer (Life Technologies, CA, USA), and integrity was tested using the RNA Nano 6000 Assay Kit of the Bioanalyzer 2100 system (Agilent Technologies, CA, USA).

RNA libraries were constructed for the 28 pigs measured in the study. A total amount of 3 μg RNA per sample was used as input material for the RNA-seq library preparations. Sequencing libraries were generated using NEBNext® Ultra™ RNA Library Prep Kit for Illumina® (NEB, USA) following the manufacturer’s recommendations and index codes were added to attribute sequences to each sample. The library preparations were sequenced on an Illumina Hiseq platform and 150 bp paired-end reads were generated.

### Differential gene expression analysis

To obtain clean reads, in-house perl scripts were used to discard adaptor sequences, reads containing ploy-N (the percentage of unknown bases is more than 10% in a read) and low quality reads from raw data (the percentage of low-quality bases of error rate > 1% is more than 50% in a read). Clean reads were then mapped to *Sus scrofa* reference genome (*Sus scrofa* 11.1, http://asia.ensembl.org/Sus_scrofa/Info/Index) using HISAT2 [30] with default parameters. Then, featureCounts tool of subread package [31] was used to count read numbers mapped to each gene.

Differential expression analysis was performed using DESeq2 (v1.18.0) [32]. It provides statistical routines for determining differential expression in gene expression data using a model based on the negative binomial distribution. The resulting *P* values were adjusted using the Benjamini and Hochberg’s approach for controlling the false discovery rate (FDR). According to the empirical studies, two criteria, i.e., the adjusted *q* value < 0.05 and fold change (FC) ≥ 2 or FC ≤ 0.5, were used as cutoffs to obtain differentially expressed (DE) genes. Genes that meet the criteria were regarded as DE genes, and their heatmap was drawn by R package pheatmap (https://cran.r-project.org/web/packages/pheatmap/).

### Filtering and functional characterization of DE genes

Pearson correlation between the expression of DE genes and their drip loss values were analyzed using R package Hmisc (https://cran.r-project.org/web/packages/Hmisc/index.html) in the data set of 28 Duroc pigs measured in this study. QTLs affecting WHC or drip loss trait were downloaded from the pig QTL database (http://www.animalgenome.org/cgi-bin/QTLdb/SS/index, Release 39, Aug 22, 2019), and compared with DE genes based on their physical position. DE genes filtered by the above two conditions were regarded as critical DE genes.

Gene Ontology (GO) and Kyoto Encyclopedia of Genes and Genomes (KEGG) pathway enrichment analyses of critical DE genes were conducted by R package “clusterProfiler” [33], which implemented hypergeometric model to select significant enriched GO term and KEGG pathway. GO terms and pathway with *P* value less than 0.01 were considered to be significantly enriched.

## Supplementary information

**Additional file 1: Table S1.** Phenotypic information of carcass and meat quality traits for the low and high samples selected for transcriptome analysis.

**Additional file 2:** **Table S2.** Summary of sequencing reads and their alignments to *Sus scrofa* reference genome.

**Additional file 3:** **Table S3.** Expressed genes detected and their normalized expression values.

**Additional file 4:** **Table S4.** Potential novel genes detected in the study.

**Additional file 5:** **Table S5.** Detailed information of DE genes detected between low and high drip loss groups.

**Additional file 6:** **Table S6.** Correlation between expressions of DE genes and their drip loss values in the 28 Duroc pigs.

**Additional file 7:** **Table S7.** Comparison of DE genes and QTLs related to drip loss.

**Additional file 8:** **Table S8.** Detailed information of critical DE genes detected between low and high drip loss groups.

**Additional file 9:** **Table S9.** Significantly enriched GO terms of the critical DE genes detected between low and high drip loss groups.

**Additional file 10:** **Table S10.** Significantly enriched pathways of the critical DE genes detected between low and high drip loss groups.

## Data Availability

All data generated in this study were included in the main article and its supplementary files. The raw data of RNA sequencing have been deposited in the National Center for Biotechnology Information Sequence Read Archive with accession No. PRJNA527944 (Available online: https://www.ncbi.nlm.nih.gov/sra/PRJNA527944).

## Abbreviations

BP: Biological process
CC: Cellular component
DE: Differentially expressed
DFD: Dark, firm and dry
EM: Extracellular matrix
GO: Gene Ontology
GWAS: Genome-wide association studies
IMF: Intramuscular fat
KEGG: Kyoto Encyclopedia of Genes and Genomes
LD: Longissimus dorsi
PPI: Protein-protein interaction
PSE: Pale, soft and exudative
QTLs: Quantitative trait loci
RNA-seq: RNA sequencing
STRING: Search Tool for the Retrieval of Interacting Genes
WHC: Water holding capacity
